# Effectiveness of comprehensive nursing care combined with an intermittent pneumatic compression device for preventing lower extremity venous thrombosis in long-term bedridden elderly patients: A prospective controlled study

**DOI:** 10.1097/MD.0000000000048601

**Published:** 2026-06-26

**Authors:** Yazhen Liu, Tingzi Zhang, Lu Zhang, Qiong Luo

**Affiliations:** a Department of Neurosurgical Spine Surgery, Ningbo Sixth Hospital, Ningbo City, Zhejiang Province, China; b Department of Internal Medicine, Ningbo Sixth Hospital, Ningbo City, Zhejiang Province, China.

**Keywords:** coagulation function, integrated care, intermittent pneumatic compression device, long-term bedridden elderly patients, lower extremity venous thrombosis, NRS score

## Abstract

To evaluate the effectiveness of comprehensive nursing care combined with an intermittent pneumatic compression (IPC) device in preventing lower extremity deep vein thrombosis (DVT) in long-term bedridden elderly patients. This prospective controlled study included 180 long-term bedridden elderly patients admitted between July 2022 and January 2024. Due to routine clinical practice, participants were not randomly assigned but allocated to control or intervention groups based on physician judgment and patient preference. The control group (n = 81) received conventional nursing care, while the intervention group (n = 99) received IPC treatment combined with comprehensive nursing care. After 2 weeks, DVT incidence, lower limb circumference differences, coagulation indices (prothrombin time, activated partial thromboplastin time, fibrinogen, D-dimer), Numerical Rating Scale pain scores, and nursing satisfaction were compared. The intervention group showed significantly greater reductions in thigh and calf circumference differences (*P* < .001). Prothrombin time and activated partial thromboplastin time were significantly prolonged, while fibrinogen and D-dimer levels were significantly reduced in the intervention group compared to controls (all *P* < .001). DVT incidence was significantly lower in the intervention group (13.13% vs 33.33%, *P* = .001). Numerical Rating Scale pain scores decreased more markedly in the intervention group (*P* < .001), and nursing satisfaction was significantly higher (94.95% vs 82.72%, *P* = .008). Comprehensive nursing care combined with IPC treatment was associated with reduced lower limb swelling, improved coagulation parameters, lower DVT risk, enhanced pain relief, and higher nursing satisfaction in long-term bedridden elderly patients.

## 1. Introduction

As the population ages, the incidence of chronic diseases among the elderly is rising annually, leading many to require prolonged bed rest. These long-term bedridden patients not only experience a diminished quality of life but are also susceptible to various complications, the most common being lower extremity deep vein thrombosis (DVT).^[1–3]^ DVT can cause swelling, pain, and increased skin temperature in the lower limbs, further impairing quality of life. More critically, it can lead to severe complications like pulmonary embolism, which carries high morbidity and mortality rates and poses a significant threat to patient safety.^[4–7]^ Consequently, the effective prevention of DVT has become a pressing clinical challenge.

Comprehensive nursing care is a patient-centered model that utilizes a multidisciplinary team to provide individualized treatment and support, addressing patients’ physical, psychological, and social needs to enhance their quality of life and promote recovery.^[8,9]^ Research indicates that this approach is particularly effective in improving outcomes for chronically ill or frail patients. It has been shown to significantly reduce complication rates, shorten hospital stays, increase patient satisfaction, and improve overall quality of life.^[10]^ In addition, comprehensive nursing care can help improve the professional quality and service level of nursing staff and promote the effective use of medical resources.^[11]^

Intermittent pneumatic compression (IPC) is a physical therapy device used for DVT prophylaxis. It functions by intermittently inflating and deflating cuffs wrapped around the legs, which massage the muscles, intermittently increase venous pressure, and promote venous return.^[12–14]^ The efficacy of IPC in preventing DVT is well-documented, with numerous systematic reviews and meta-analyses confirming that its use significantly reduces the incidence of postoperative DVT.^[14–16]^ Another study of postoperative femoral neck fractures also found that the combination of IPC significantly reduced the risk of DVT compared with low molecular weight heparin.^[17]^ In addition, IPC is simple, noninvasive, and easily accessible, making it suitable for patients who are bedridden or have limited mobility.^[18,19]^ This study aims to provide a scientific basis for reducing the incidence of complications such as DVT by comparatively analyzing the differences in the effects of comprehensive nursing care combined with IPC devices and conventional care.

## 2. Materials and methods

### 2.1. Study population

This prospective controlled study included 180 long-term bedridden elderly patients who were admitted to our hospital from July 2022 to January 2024. Due to the nature of the intervention and routine clinical practice, participants were not randomly assigned. Instead, they were allocated to either the control group or the observation group based on the intervention they received, as determined by the attending physician’s clinical judgment and patient preference following a detailed discussion of the potential risks and benefits of each approach. This non-randomized allocation is a key limitation of the study design and is addressed as such in the discussion. The control group comprised 81 patients who received conventional nursing interventions, while the observation group included 99 patients who received IPC device treatment in combination with comprehensive nursing care. The study was approved by the Ethics Committee of Ningbo Sixth Hospital and strictly adhered to the Declaration of Helsinki. All study subjects and their families provided written informed consent to participate. The ethics committee approved the consent procedure, and all participants were informed that they could withdraw from the study at any time without affecting their medical care. All 180 enrolled patients completed the 2-week intervention and follow-up period with complete data for all outcome measures; therefore, there were no dropouts or missing data to address.

The sample size was estimated using PASS 15.0 software (NCSS, LLC). Based on a previous pilot study, the expected incidence of DVT in the control group was 35% and in the intervention group was 15%. To detect this 20% absolute difference with a two-sided test, an α of 0.05, and a power (1 − β) of 80%, a minimum of 73 patients per group was required. Accounting for a potential 10% dropout rate, the target enrollment was set at 81 patients for the control group and 99 for the intervention group.

### 2.2. Inclusion criteria

The inclusion criteria were: age between 60 and 80 years; expected duration of being bedridden of more than 1 month; absence of DVT at the time of enrollment, confirmed by ultrasound; and provision of written informed consent by the patient or their legal representative.

### 2.3. Exclusion criteria

The exclusion criteria were: incomplete clinical data; severe dysfunction of vital organs such as the heart, liver, or kidneys; unstable vital signs; impaired consciousness or mental disorders that would preclude participation in the study; and concomitant hematologic or coagulation system disorders.

### 2.4. Data collection

General clinical data were collected from all patients at admission, including age, gender, body mass index (BMI), history of diabetes mellitus, history of hypertension, reason for being bedridden (lower limb fracture, stroke hemiplegia, or other), and the duration of being bedridden.

### 2.5. Treatment

Patients in the control group received routine nursing interventions. Healthcare personnel closely monitored and regularly recorded vital signs (temperature, blood pressure, heart rate, respiration) to promptly detect any changes in their condition. Postural care included assisting patients to turn over every 2 hours and massaging their lower limbs. The lower limbs were elevated by 20° to 30°, and patients were instructed to avoid excessive limb flexion. When their physical condition allowed, they were encouraged to get out of bed and sit in a wheelchair for 15 to 30 minutes. Efforts were made to protect venous vessels and to avoid using vasopressor-stimulating medications whenever possible. Of note, neither group received routine pharmacologic thromboprophylaxis (e.g., low molecular weight heparin) during the study period, as the primary focus was on non-pharmacologic prevention strategies. Patients with preexisting anticoagulant therapy for other indications were excluded from the study.

Patients in the intervention group received the comprehensive nursing care protocol detailed below, in addition to the standard vital sign monitoring and basic care provided to the control group. In contrast to conventional care, which focuses primarily on basic monitoring and support, this comprehensive nursing care protocol incorporates structured psychological support, personalized dietary guidance, and staged rehabilitation training to address the multifaceted needs of long-term bedridden patients. The key, measurable components of this protocol were: specialized lower limb monitoring: daily measurement and comparison of the difference in circumference between the lower limbs and the lower legs at the same anatomical landmark to detect early signs of DVT. Skin color, temperature, and degree of swelling were also assessed daily. Structured psychological care: nursing staff conducted 15 to 20-minute structured communication sessions with patients 3 times per week. These sessions involved active listening, addressing patient concerns, and providing education on disease-related knowledge, treatment progress, and successful rehabilitation cases to alleviate anxiety and depression. Personalized dietary guidance: a registered dietitian formulated personalized meal plans for each patient. The plans emphasized a high-fiber diet (aiming for 25–30 g of fiber per day) from fresh vegetables, fruits, and whole grains to prevent constipation. They also restricted fat and cholesterol intake by recommending lean meats, fish, and legumes to manage blood viscosity. Staged rehabilitation training: Phase 1 (early bedridden, Days 1–7): patients were assisted with active in-bed exercises, including deep breathing (10 reps), ankle pumps (dorsiflexion/plantarflexion, 15 reps per foot), and knee flexion/extension (10 reps per leg). Each exercise was performed in 3 sets per day. Phase 2 (adaptation, Days 8–14): as tolerance improved, exercises were progressed to bedside sitting, standing balance training, and short-distance walking with assistance for 10 to 20 minutes per session, 2 to 3 times a day, based on individual patient tolerance.

On this basis, IPC was added to the observation group.^[20]^ The Korean Daesung DVT-2600 pressure anti-embolism pump was selected, and the appropriate airbag pressure was adjusted according to the thickness, fatness and thinness of the patient’s legs. Generally, the initial pressure was set at 30 to 40 mm Hg, and then gradually increased to 60 to 80 mm Hg according to the patient’s tolerance, which was suitable for the patient’s comfort and effective promotion of blood flow. The inflation time is set at 30 to 60 seconds to ensure that the airbag is fully inflated and produces moderate compression on the muscles and blood vessels of the lower limbs; the deflation time is set at 20 to 40 seconds to relax the blood vessels and restore blood perfusion. The treatment time is 20 to 30 minutes for 2 times a day, and 2 consecutive weeks of treatment is 1 course of treatment.

### 2.6. Evaluation indexes

(1) Record the circumference of the lower limbs of the 2 groups of patients before and after the intervention, and measure the difference between the circumferences of the large and small legs of the enrolled patients using a tape measure at a fixed position before and after 2 weeks of the intervention in order to evaluate the degree of swelling of their lower limbs.
(2) Compare the coagulation function indexes of the 2 groups of patients before and after the intervention. Before and after the intervention, fasting venous blood was collected from both groups in a volume of 5 mL, and prothrombin time (PT), activated partial thromboplastin time (APTT), fibrinogen (FIB), and D-dimer (D-D) were detected.
(3) To compare the incidence of DVT between the 2 groups. The occurrence of lower extremity DVT was assessed at baseline and after the 2-week intervention period. DVT was diagnosed using color Doppler duplex ultrasound (Philips EPIQ 7C) performed by a single, experienced radiologist who was strictly blinded to the patients’ group allocation. The diagnostic criteria for acute DVT were: visualization of a solid echogenic thrombus within the deep vein lumen; lack of complete compressibility of the vein upon gentle pressure with the ultrasound probe, which is the most definitive diagnostic feature; and absence of spontaneous or phasic Doppler flow, or presence of nonpulsatile, continuous flow distal to the thrombus. All patients underwent systematic ultrasound screening at baseline and again after the 2-week intervention period.
(4) Before and after the intervention, the patients’ pain level was assessed using the Numerical Rating Scale (NRS) score. The NRS score totaled 10 points, with higher scores indicating more pain for the patients. Pain was assessed using the NRS by an independent research nurse who was blinded to the patients’ group allocation.
(5) Nursing satisfaction was assessed using a self-developed questionnaire designed to evaluate patient satisfaction with the nursing care received. The questionnaire was developed by a panel of 3 senior nursing specialists based on a literature review of patient satisfaction instruments and clinical experience in geriatric care. Its content validity was established through expert review to ensure the items comprehensively covered the key dimensions of nursing care relevant to this patient population. The final questionnaire consisted of 20 items covering 3 dimensions: quality of nursing implementation (8 items), nursing service attitude (6 items), and patient education and communication (6 items). Each item was scored on a 5-point Likert scale (1 = very dissatisfied to 5 = very satisfied), with total scores ranging from 20 to 100. The total score was categorized as follows: ≥90 = satisfied, 60 to 89 = basically satisfied, and <60 = dissatisfied. The nursing satisfaction rate was calculated as the percentage of patients who were either “satisfied” or “basically satisfied.” The questionnaire demonstrated good internal consistency in this study sample, with a Cronbach α coefficient of 0.87. An example item is, “The nursing staff was attentive to my concerns and provided clear explanations about my care.”

### 2.7. Statistical analysis

Statistical analysis was performed using SPSS 26.0 (IBM Corp.) and GraphPad Prism 9.0 (GraphPad Software Inc.). The normality of continuous data was tested using the Shapiro–Wilk test. The normality of continuous data was tested using the Shapiro–Wilk test. Data following a normal distribution were presented as mean ± standard deviation and analyzed using the paired *t* test for within-group comparisons and the independent samples *t* test for between-group comparisons. Data not following a normal distribution were presented as median with interquartile range (*M* [Q1, Q3]) and analyzed using the Wilcoxon signed-rank test for within-group comparisons and the Mann–Whitney *U* test for between-group comparisons. The corresponding test statistics (*t* for parametric tests, *Z* for nonparametric tests) are reported alongside the *P* values in the tables. Categorical data were expressed as frequencies and percentages (n [%]) and analyzed using the chi-square test or Fisher exact test, as appropriate. Statistical analysis was performed using SPSS 26.0 (IBM Corp.) and GraphPad Prism 9.0 (GraphPad Software Inc.). The normality of continuous data was tested using the Shapiro–Wilk test. Data following a normal distribution were presented as mean ± standard deviation and analyzed using the paired *t* test for within-group comparisons and the independent samples *t* test for between-group comparisons. Data not following a normal distribution were presented as median with interquartile range (*M* [Q1, Q3]) and analyzed using the Wilcoxon signed-rank test for within-group comparisons and the Mann–Whitney *U* test for between-group comparisons. The corresponding test statistics (*t* for parametric tests, *Z* for nonparametric tests) are reported alongside the *P* values in the tables. Categorical data were expressed as frequencies and percentages (n [%]) and analyzed using the chi-square test or Fisher exact test, as appropriate. To control for potential confounders given the non-randomized design, a multivariable logistic regression analysis was performed to identify independent predictors of DVT (the primary outcome). Variables with a *P* value <.10 in the univariate analysis, along with clinically relevant factors such as age, gender, and BMI, were entered into the regression model. Results are reported as adjusted odds ratios with 95% confidence intervals. A two-tailed *P* value of <.05 was considered statistically significant for all analyses.

## 3. Results

### 3.1. Clinical baseline characteristics of the enrolled population

In this study, 180 long-term bedridden elderly patients were included as research subjects, and were divided into a control group (n = 81) and an observation group (n = 99) according to the different intervention programs. The clinical baseline data of the 2 groups were compared and analyzed, as shown in Table 1, there was no significant difference between the 2 groups in terms of age, gender, BMI, history of diabetes mellitus, history of hypertension, reason for bedridden and bedridden time (*P* > .05), and the 2 groups were comparable.

**Table 1:** Clinical baseline information of the enrolled population.

Parameter	Control group (n = 81)	Observation group (n = 99)	Test statistic (*t*/χ^2^)	*P* value
Age (yr), mean ± SD	70.19 ± 2.62	70.67 ± 3.10	1.110	.269
Sex, n (%)				
Man	50 (61.73%)	63 (63.64%)	0.069	.792
Women	31 (38.27%)	36 (36.36%)		
BMI (kg/m^2^), mean ± SD	24.39 ± 1.73	24.75 ± 1.61	1.458	.147
History of diabetes, n (%)	14 (17.28%)	19 (19.19%)	0.108	.742
History of hypertension, n (%)	29 (35.80%)	38 (38.38%)	0.127	.722
Reason for being bedridden, n (%)	29.75 ± 5.23	30.94 ± 6.08	1.322	.188
Lower limb fracture	29 (35.80%)	34 (34.34%)	0.058	.971
Hemiplegia	33 (40.74%)	42 (42.42%)		
Other	19 (23.46%)	23 (23.23%)		
Duration of being bedridden (d), mean ± SD	43.22 ± 6.38	42.11 ± 6.49	1.152	.251

Measured data were expressed as mean ± SD and analyzed by unpaired *t* test. Count data were expressed as number of cases and percentage and analyzed by chi-square test, and the difference was statistically significant at *P* < .05.

BMI = body mass index, SD = standard deviation.

### 3.2. Comparison of the difference between the circumference of the greater and lesser leg before and after the intervention in the 2 groups of patients

The degree of lower limb swelling was assessed by measuring the difference between the patients’ thigh and calf circumferences. Before intervention, there was no statistically significant difference in the difference between the large and small leg circumferential diameters of the 2 groups of patients, which was comparable (all *P* > .05; Table 2). Compared with the pre-intervention period, the difference between the circumferential diameter of the large and small legs of the 2 groups of patients decreased after 2 weeks of intervention (both *P* < .001), and the difference between the circumferential diameter of the large and small legs of the observation group was significantly smaller than that of the control group (both *P* < .001; Table 2). The above results indicate that the positive effect of comprehensive nursing care combined with IPC treatment on reducing lower limb swelling is conducive to promoting venous return and reducing the risk of thrombosis.

**Table 2:** Comparison of the difference between the circumference of the thigh and calf in the 2 groups of patients.

Parameter	Group	n	Pre-intervention (cm)	Post-intervention (cm)	Within-group *t*	Within-group *P*
Thigh circumference difference	Control	81	2.58 ± 0.28	1.96 ± 0.27	14.450	<.001
	Intervention	99	2.61 ± 0.32	1.70 ± 0.28	21.030	<.001
	Between-group *t*		0.679	6.220		
	Between-group *P*		.498	<.001		
Calf circumference difference	Control	81	1.79 ± 0.14	1.15 ± 0.22	22.150	<.001
	Intervention	99	1.80 ± 0.20	0.96 ± 0.18	29.180	<.001
	Between-group *t*		0.590	6.522		
	Between-group *P*		.556	<.001		

Data are presented as mean ± SD. Within-group comparisons were performed using paired *t* tests. Between-group comparisons were performed using independent samples *t* tests. *P* < .05 was considered statistically significant.

SD = standard deviation.

### 3.3. Comparison of coagulation function before and after intervention in 2 groups of patients

We compared the levels of coagulation function (PT, APTT, FIB, and D-D) before and after the intervention in the 2 groups of patients, and found that, before the intervention, there was no statistically significant difference in the levels of coagulation function (PT, APTT, FIB, and D-D) in the 2 groups (both *P* > .05; Table 3); after the intervention, the levels of PT and APTT in the 2 groups of patients were significantly higher, and the levels of FIB and D-D were significantly decreased (all *P* < .001). In addition, compared with the control group, the PT and APTT levels of patients in the observation group were increased, and the FIB and D-D levels were significantly reduced (all *P* < .001; Table 3). The above results indicate that comprehensive nursing care combined with IPC treatment has a positive regulatory effect on the coagulation function of elderly patients and can synergistically improve their hypercoagulable state.

**Table 3:** Comparison of coagulation function before and after intervention in 2 groups of patients.

Parameter	Group	n	Pre-intervention	Post-intervention	Test statistic	*P* value
PT (s), median (Q1, Q3)	Control	81	10 (10, 11)	12 (11, 12)	*Z* = −6.184	<.001
	Intervention	99	10 (10, 11)	12 (11, 13)	*Z* = −8.101	<.001
	Between-group		*Z* = −0.321, *P* = .749	*Z* = −2.858, *P* = .004		
APTT (s), mean ± SD	Control	81	26.77 ± 2.69	31.88 ± 3.29	*t* = 11.160	<.001
	Intervention	99	26.91 ± 2.84	33.40 ± 3.53	*t* = 14.990	<.001
	Between-group		*t* = 0.346, *P* = .730	*t* = 2.976, *P* = .003		
FIB (g/L), mean ± SD	Control	81	4.56 ± 0.77	3.56 ± 0.62	*t* = 9.006	<.001
	Intervention	99	4.61 ± 0.86	3.12 ± 0.70	*t* = 13.590	<.001
	Between-group		*t* = 0.447, *P* = .656	*t* = 4.462, *P* < .001		
D-D (μg/L), mean ± SD	Control	81	0.50 ± 0.07	0.40 ± 0.09	*t* = 7.484	<.001
	Intervention	99	0.51 ± 0.09	0.35 ± 0.08	*t* = 15.160	<.001
	Between-group		*t* = 0.738, *P* = .461	*t* = 4.088, *P* < .001		

Data are presented as mean ± SD for normally distributed variables (APTT, FIB, and D-D) and as median (Q1, Q3) for non-normally distributed variables (PT), as determined by the Shapiro–Wilk test. For normally distributed data, the paired *t* test was used for within-group comparisons and the independent samples *t* test for between-group comparisons, with the *t*-statistic reported. For non-normally distributed data, the Wilcoxon signed-rank test was used for within-group comparisons and the Mann–Whitney *U* test for between-group comparisons, with the *Z*-statistic reported. Differences were considered statistically significant at *P* < .05.

APTT = activated partial thromboplastin time, D-D = D-dimer, FIB = fibrinogen, PT = prothrombin time, SD = standard deviation.

### 3.4. Comparison of the incidence of lower extremity DVT between the 2 groups of patients

After the 2-week intervention, 27 patients (33.33%) in the control group developed lower extremity DVT compared to only 13 patients (13.13%) in the observation group. This difference was statistically significant (χ^2^ = 10.521, *P* = .001), indicating that comprehensive nursing care combined with IPC treatment is significantly more effective in reducing the risk of DVT in this patient population. To further account for the non-randomized design, a multivariable logistic regression analysis was conducted to assess the independent effect of the intervention on DVT risk. After adjusting for age, gender, BMI, comorbid conditions (diabetes and hypertension), reason for being bedridden, and baseline D-dimer levels, the intervention group remained independently associated with a significantly lower odds of DVT compared to the control group (adjusted odds ratios = 0.29, 95% confidence interval = 0.13–0.61, *P* = .001). The comparison of DVT incidence between the 2 groups showed that the intervention group had a significantly lower incidence of DVT (13.13%) compared to the control group (33.33%; *P* = .001).

### 3.5. Comparison of NRS scores before and after intervention in 2 groups of patients

The NRS score was used to assess the pain level of the 2 groups of patients. It was found that there was no statistically significant difference in NRS scores between the 2 groups of patients before intervention (*P* = .201; Table 4). The NRS scores of the 2 groups of patients decreased significantly after the intervention (both *P* < .001), and the NRS scores of the observation group were significantly lower than those of the control group (*P* < .001; Table 4). The above results indicate the significant role of comprehensive nursing care combined with IPC treatment in relieving patients’ pain.

**Table 4:** Comparison of NRS scores before and after intervention in the 2 groups of patients.

Group	n	Pre-intervention	Post-intervention	Within-group *Z*	Within-group *P*	Group
Control	81	4 (4, 5)	3 (3, 4)	−6.824	<.001	Control
Intervention	99	4 (4, 5)	3 (2, 3)	−8.458	<.001	Intervention
Between-group *Z*		−1.295	−4.594			
Between-group *P*		.201	<.001			

Measurements that do not conform to normal distribution are expressed in quartiles, before and after treatment using the Wilcoxon signed-rank test, and the Mann–Whitney *U* test between groups. Statistically significant differences were indicated by *P* < .05.

NRS = Numerical Rating Scale.

### 3.6. Comparison of nursing satisfaction rate between the 2 groups of patients

After 2 weeks of intervention, the nursing satisfaction rate of patients in the 2 groups was compared. It was found that the nursing care satisfaction rate of patients in the control group was 82.72%, and the nursing care satisfaction rate of the observation group was 94.95%. Compared with the control group, the nursing satisfaction rate of the observation group was significantly higher, and the difference was statistically significant (*P* = .008; Table 5). The above results show that indicate that comprehensive nursing care combined with IPC treatment has a significant effect in improving patient care satisfaction.

**Table 5:** Comparison of satisfaction rate of nursing care between 2 groups of patients.

Group	n	Satisfied, n (%)	Basically satisfied, n (%)	Dissatisfied, n (%)	Satisfaction rate, n (%)
Control	81	23 (28.4)	44 (54.3)	14 (17.3)	67 (82.7)
Intervention	99	36 (36.4)	58 (58.6)	5 (5.0)	94 (94.9)
χ^2^					7.062
*P* value					.008

Count data were expressed as number of cases and percentage using chi-square test, with *P* < .05 indicating a statistically significant difference.

## 4. Discussion

Lower extremity venous thrombosis is a common postoperative complication, and its incidence has been increasing in recent years, significantly impacting patients’ quality of life.^[21–23]^

This study demonstrates the multifaceted benefits of combining comprehensive nursing care with IPC for DVT prophylaxis. While conventional care provides essential monitoring and support, it may not fully address the complex needs of long-term bedridden patients. In contrast, comprehensive nursing care offers a broader, more targeted range of interventions. Its holistic approach, incorporating structured psychological support, personalized dietary guidance, and staged rehabilitation training, appears to be instrumental in not only improving clinical indicators like coagulation function but also in enhancing patient satisfaction and reducing DVT incidence.^[20,24]^ Previous studies have also confirmed the role of IPC in promoting venous return.^[25–27]^ A systematic evaluation noted that IPC significantly reduced lower limb edema in postoperative patients.^[28–30]^ The results of this study demonstrated that the reduction in the difference between thigh and calf circumferences was significantly greater in the observation group than in the control group. This finding suggests that IPC effectively promotes lower limb blood circulation and reduces edema by periodically applying pressure to the limbs.

A notable finding was the significant modulation of coagulation parameters in the intervention group. The prolongation of PT and APTT, along with reduced FIB and D-D levels, indicates a shift away from a hypercoagulable state-a well-established risk factor for venous stasis and thrombosis.^[31,32]^ The dietary component of the comprehensive nursing care, which emphasized a high-fiber, low-fat diet, may have contributed to this by influencing blood viscosity and the substrate availability for coagulation factor synthesis.^[33–35]^ Concurrently, IPC is thought to exert its antithrombotic effects through multiple mechanisms, including enhancing venous return to prevent stasis and, as some studies suggest, increasing systemic fibrinolytic activity by stimulating endothelial release of tissue plasminogen activator.^[14,36,37]^ Therefore, the synergistic effect of comprehensive nursing care and IPC likely provides a dual benefit: 1 pathway optimizes the internal milieu (e.g., through diet), while the other provides a direct mechanical stimulus for circulation and fibrinolysis, collectively leading to the superior outcomes observed. In this study, the incidence of DVT was significantly lower in the observation group than in the control group. This finding aligns with previous research showing that comprehensive nursing care combined with IPC significantly reduces the risk of perioperative DVT in patients with ovarian cancer compared to conventional care alone.^[20]^ These results reinforce the role of IPC as an effective prophylactic measure for DVT.^[13,27,38]^ It is important to note that neither group received routine pharmacologic thromboprophylaxis during the study period, as patients on anticoagulants were excluded. Therefore, the observed improvements in coagulation parameters in the intervention group can be attributed to the combined effects of comprehensive nursing care and IPC, rather than concurrent anticoagulant medication. The dietary modifications aimed at reducing fat intake may have contributed to reduced substrate availability for coagulation factor synthesis, while IPC may have enhanced fibrinolytic activity, collectively leading to the favorable coagulation profile observed in the intervention group.

Pain is an important factor affecting patients’ recovery and quality of life, and effective pain management is essential for improving patient satisfaction.^[39]^ Comprehensive nursing care can effectively alleviate pain in elderly patients undergoing surgery under general anesthesia.^[40]^ Studies have shown that IPC can effectively reduce patients’ pain by improving blood circulation and reducing tissue edema.^[28]^ In this study, comprehensive nursing care combined with IPC significantly relieved patients’ pain, thereby enhancing patient satisfaction. A survey of hospitalized patients found that high-quality nursing care can significantly improve patient satisfaction and contribute to a better doctor–patient relationship.^[41,42]^

Beyond statistical significance, the observed differences between groups are clinically meaningful. The 20.2% absolute reduction in DVT incidence (from 33.33% to 13.13%) corresponds to a number needed to treat of approximately 5, meaning that only 5 patients need to receive the combined intervention to prevent 1 additional DVT event-a clinically worthwhile effect. The reduction in limb circumference differences (0.26 cm for thigh, 0.19 cm for calf) represents a meaningful decrease in edema that can improve patient comfort and mobility. The prolongation of PT and APTT by 1 to 2 seconds, while modest, indicates a shift away from a hypercoagulable state that may reduce thrombotic risk. The 0.5 to 1 point reduction in NRS pain scores on a 10-point scale, though small, contributed to improved patient satisfaction and quality of life. Furthermore, the 12.2% absolute increase in nursing satisfaction rate (from 82.7% to 94.9%) reflects a substantial improvement in patient experience. These clinically relevant improvements support the value of implementing this combined intervention in clinical practice.

In conclusion, the combination of comprehensive nursing care and IPC treatment is a highly effective strategy for preventing DVT in long-term bedridden elderly patients. This integrated approach significantly reduces lower limb swelling, ameliorates hypercoagulability, alleviates pain, and ultimately lowers the risk of thrombosis, leading to improved patient recovery, enhanced quality of life, and higher satisfaction with nursing care.

This study has several limitations that should be acknowledged. First, the non-randomized design is a major limitation, as it introduces the potential for selection bias despite the comparability of baseline characteristics. Furthermore, the intervention group received a more intensive nursing protocol in addition to IPC, while the control group received only conventional care. This unequal intensity of care makes it difficult to isolate the specific effect of IPC from the broader effects of increased attention, psychological support, dietary guidance, and rehabilitation training. Unmeasured confounding variables related to these additional interventions may have influenced the observed outcomes. Unmeasured confounding variables may have influenced the results. Second, this was a single-center study with a relatively modest sample size, which may limit the generalizability of our findings to other settings or populations. Third, the follow-up period was short (2 weeks), preventing any assessment of the long-term efficacy or safety of the intervention, including the potential for late-onset DVT or recurrence. Fourth, while the nursing satisfaction questionnaire demonstrated good internal consistency (Cronbach α = 0.87), it was a non-standardized, self-developed tool, which may introduce bias and limits comparability with other studies. Furthermore, because the intervention group received more intensive nursing care, including structured psychological support and personalized attention, satisfaction scores in this group are highly susceptible to bias related to increased caregiver interaction rather than the specific effects of IPC or the comprehensive nursing protocol. Patients may have rated their satisfaction higher simply due to the additional attention received, rather than the clinical effectiveness of the intervention. This potential bias should be considered when interpreting the satisfaction outcomes. Finally, the lack of a cost-effectiveness analysis means we cannot comment on the economic viability of implementing this combined intervention more broadly.

Future research should address these limitations by employing a multicenter, randomized controlled trial design with a larger, more diverse sample. Such studies should include longer follow-up periods to assess long-term outcomes and should incorporate blinded outcome assessors for all endpoints. In addition, the use of validated, standardized instruments for assessing patient-reported outcomes like pain and satisfaction, alongside formal cost-effectiveness analyses, would provide more robust and generalizable evidence to guide clinical practice and health policy.

## Acknowledgments

The authors would like to thank the nursing staff of the Department of Geriatric Nursing at Ningbo Sixth Hospital for their support and assistance in conducting this study. All individuals named in this section have provided written consent to be acknowledged.

## Author contributions

**Formal analysis:** Yazhen Liu.

**Project administration:** Yazhen Liu, Lu Zhang.

**Writing – review & editing:** Yazhen Liu, Qiong Luo.

**Software:** Tingzi Zhang.

**Validation:** Tingzi Zhang.

**Writing – original draft:** Tingzi Zhang, Lu Zhang.

**Methodology:** Lu Zhang.

**Conceptualization:** Qiong Luo.

**Visualization:** Qiong Luo.
